# Multichart Schemes for Detecting Changes in Disease Incidence

**DOI:** 10.1155/2020/7267801

**Published:** 2020-05-15

**Authors:** Gideon Mensah Engmann, Dong Han

**Affiliations:** ^1^School of Mathematical Sciences, Shanghai Jiao Tong University, 200240 Shanghai, China; ^2^Department of Statistics, University for Development Studies, Navrongo, Ghana

## Abstract

Several methods have been proposed in open literatures for detecting changes in disease outbreak or incidence. Most of these methods are likelihood-based as well as the direct application of Shewhart, CUSUM and EWMA schemes. We use CUSUM, EWMA and EWMA-CUSUM multi-chart schemes to detect changes in disease incidence. Multi-chart is a combination of several single charts that detects changes in a process and have been shown to have elegant properties in the sense that they are fast in detecting changes in a process as well as being computationally less expensive. Simulation results show that the multi-CUSUM chart is faster than EWMA and EWMA-CUSUM multi-charts in detecting shifts in the rate parameter. A real illustration with health data is used to demonstrate the efficiency of the schemes.

## 1. Introduction

In this era of bioterrorism, outbreak of diseases and surge in disease incidence; statisticians, epidemiologists, informaticians and surveillance scientists are designing algorithms to detect changes in disease occurrence or outbreak in order to avert any possible public health pandemonium. Most of these models or algorithms are modifications of the statistical process control (SPC) schemes, namely Shewhart, CUmulative SUM (CUSUM) and Exponentially Weighted Moving Average (EWMA) statistics.

Biosurveillance in the context of human health (health surveillance) is a term for the science and practice of managing health-related data and information for early warning of threats and hazards, early detection of events and rapid characterization of the event so that effective actions can be taken to mitigate adverse health effects [1]. Biosurveillance systems have two main purposes: to support health situational awareness and for early event/outbreak detection. In the past two or three decades, many biosurveillance systems have been developed. Bravata et al. [2] in their review identified 115 health surveillance systems and 9 syndromic surveillance systems. Most of these surveillance systems have been developed and are in use in countries like US, UK, China and Japan among others.

Statistical methods or algorithms have been widely applied to solve biosurveillance problems. These statistical methods or algorithms for monitoring bioterrorism, incidence, or outbreak of diseases can be categorized into temporal (see, for example, Reis [3] and Brookmeyer and Stroup [4]), spatio (Waller and Gotway [5] and Lawson and Kleinman [6]), spatio-temporal (Diggle [7] and Fricker [8]), multivariate temporal (see Vial [9]), multivariate spatial monitoring (see, for example, Corberán-Vallet [10]), multivariate spatio-temporal (Quick et al. [11]), and Bayesian (Tzala [12]). Multivariate monitoring methods are extension of the univariate methods.

Several methods have been proposed in literatures, for example, Farrington et al. [13] proposed a robust statistical algorithm to process weekly reports of infections received at the Communicable Disease Surveillance Centre. The algorithm calculates suitable thresholds and organisms exceeding their thresholds are then flagged for further investigation. Le Strat and Carrat [14] proposed a hidden markov model to monitor epidemiologic surveillance data. A rule-based method was also proposed by Wong [15] to solve a surveillance classification problem. Many Point Process Models (PPM) were also discussed by Brookmeyer and Stroup [4]. Shmueli et al. [16] proposed a wavelet-based automated algorithm for detecting disease outbreaks in temporal syndromic data. Their method improves upon the Goldenberg et al. [17] algorithm on a diverse set of real syndromic data from multiple data sources and multiple geographical locations. Sebastiani et al. [18] proposed a Bayesian dynamic model to monitor influenza surveillance data. They integrated different data sources into a dynamic model, which identified in-children and infants pediatric emergency departments with respiratory syndromes as an early indicator of impending influenza morbidity and mortality. Their findings show that dynamic Bayesian networks could be suitable modeling tools for developing epidemic surveillance systems. Forsberg et al. [19] also proposed the so-called distance-based method where they assessed possible disease clusters based on *M*-statistic on the distribution of the pairwise distance between cases. Fricker [20] and Joner et al. [21] also considered Directional Multivariate Exponentially Weighted Moving Average (DMEWMA) and Multivariate CUmulative SUM (MCUSUM) schemes. Fricker et al. [22] proposed CUSUM-based methods with adaptive regression. Fricker and Chang [23] considered Repeated Two-sample Rank (RTR)-based methods. Their proposed method is spatio-temporal and can subsequently be used to track the spread of an outbreak. Lu et al. [24] proposed the Markov switching model to detect disease outbreak. Cowling et al. [25] developed a statistical algorithm using sentinel surveillance data for early detection of the annual influenza peak season in Hong Kong. Bédubourg and Le Strat [26] compared and evaluated using the simulation of several statistical methods for early temporal detection of outbreaks. Even though many methods have been developed and proposed in literature, there is still the need for more concerted efforts to develop and improve methods that will detect changes in disease incidence or outbreak and monitor bioterrorism.

Many researchers have used a Poisson process to monitor changes in disease outbreak or incidence. Rossi et al. [27] used CUSUM charts to monitor changes in disease occurrence after the transformation of the Poisson data into approximately normal random variables. Mei et al. [28] proposed a weighted CUSUM chart placing more weight on recent observations and compared their method with other common CUSUM techniques. Jiang et al. [29] compared the performance of several CUSUM methods subject to Poisson distribution. Richards et al. [30] proposed an invariant Poisson control charting scheme and applied it to monitor the number of emergency arrivals observed at the Baltimore Veterans Affairs Medical Center. Most of these models or schemes monitor one variable or disease at a time. Even in cases where two or more diseases are monitored, MCUSUM and MEWMA schemes have been used widely. Multi-charts have been shown in literature to be very powerful to detect changes in random events. They are different from MCUSUM (see Crosier [31], Golosnoy [32], and Raji et al. [33]), MEWMA (see Lowry et al. [34], Hussain et al. [35] and Ajadi and Riaz [36]), and multi-hypothesis testing (see Baum and Veeravalli [37] and Lai [38]) in terms of methodology. For example, multi-chart schemes can tell which of the charts triggered detection, a property that falls short of multivariate charts (MCUSUM and MEWMA). Multi-chart consists of several single charts with different reference values that are used simultaneously to detect and monitor process changes. CUSUM multi-chart scheme has been shown to be more efficient than the EWMA multi-chart scheme in detecting changes in a random process (Han et al. [39]). Multi-chart schemes have elegant properties in the sense that they are fast in detecting changes in a process and computationally less expensive than sister charts like Generalized Exponential Weighted Moving Average (GEWMA) by Han and Tsung [40] and the CUSUM-like control chart by Siegmund and Venkatraman [41].

Multi-chart schemes have rarely been used in the field of biosurveillance and health monitoring. A wealth of research is ongoing in disease surveillance and these methods are implemented in health surveillance systems to detect abnormal changes in disease occurrences. The ability to detect abnormal changes in disease occurrence is of uttermost concern to the public health workers for them to trigger public awareness and education. It is in this light that we applied the methodology of multi-chart schemes to detect changes in disease incidence and also evaluate the efficiency of the methods. Many researchers have proposed charting performance indices (for example, Overall Charting Performance Index (OCPI), Relative Mean Index (RMI) among others) to evaluate the performance of CUSUM and EWMA schemes. In the computation of these indices, we need the optimal *ARL* (*ARL*^∗^) which is found subject to normal distribution (continuous distribution). The CUSUM *ARLs* with reference values as charting statistic subject to Poisson distribution are not optimal as we are dealing with discrete distribution; hence, we also propose new measures (called Expectation of the Time for Detecting mean shifts (ETD) and Expectation of the Time for Detecting mean shifts with Equal weights of shifts (ETDE)) to evaluate the efficiency of the schemes.

Basically, the objectives of this study are to monitor tuberculosis disease based on multi-chart schemes and also evaluate the efficiency of the methods using a new performance index. Generally, we only know the possible postchange region but rarely know the exact magnitude of mean shift of a process before it is detected; we therefore use a range of known shifts in the rate parameter. The main contribution of our paper is as follows: we present a new performance index measure to evaluate the performance of the charts.

The article is organized as follows: materials and methods are presented in section 2, subsection 2.1, presents the multi-chart schemes subject to Poisson distribution for detecting changes in disease incidence.  Subsection 2.2 presents the performance index measures, while subsection 2.3 gives a theoretical performance comparison of the multi-chart schemes with that of single charts and subsection 2.4 gives the procedural description of multi-chart schemes. Results and discussion are presented in section 3, where these theoretical results are compared by numerical simulations in subsections 3.1, 3.2, 3.3, and 3.4, while subsection 3.5 gives a real example based on tuberculosis data from Ghana.  Section 4 concludes and gives remarks.

## 2. Materials and Methods

### 2.1. Multi-chart Schemes

Generally in health care monitoring, the observations are counts and let's assume they follow the Poisson distribution. The Poisson distribution is usually used to describe the number of events that occurred in a unit time interval or within a unit space.

Let's assume *X*_*i*_~*Poisson*(*λ*), where *λ* is the average count of a disease occurring in a week or in a month. Usually, at some time period *ν*, the probability distribution of *X*_*i*_ changes from *Poisson*(*λ*_0_) to *Poisson*(*λ*_*ν*_). We generally refer to *ν* as a change point. In general, *ν* = 1, 2, 3, ⋯, ∞, but in this article, we assume *ν* = 1, which means the first time there is a change in distribution. Intuitively, the mean of *X*_*i*_ undergoes a shift of size *λ*_*i*_(*λ*_0_), where *λ*_0_ is known and assumed to be 1. In biosurveillance problems or health surveillance, we normally monitor for upward change in distribution, since the increment in disease counts pose challenges to the public health workers. For the Poisson distribution, the mean is equal to the variance; hence, developing a chart to monitor the mean jointly monitors both the mean and the variance simultaneously.

Mathematically, the prechange distribution with mean (*λ*_0_ = 1) is given by

(1) $$\begin{array}{c} X_{1},X_{2},\cdots ,X_{\nu -1}\text{\textasciitilde{}}P_{\lambda_{0}}\left(X_{k}\right)=\frac{\lambda_{0}^{x_{k}}e^{-\lambda_{0}}}{x_{k}!} \end{array}$$

And also the postchange distribution with mean (*λ* ≠ 1) is given by

(2) $$\begin{array}{c} X_{\nu },X_{\nu +1},\cdots ,X_{n}\text{\textasciitilde{}}P_{\lambda }\left(X_{k}\right)=\frac{\lambda^{x_{k}}e^{-\lambda }}{x_{k}!} \end{array}$$

The log-likelihood ratio (*γ*_*k*_) for *ν* ≤ *k* ≤ *n* is given by

(3) $$\begin{array}{c} \gamma_{k}=\ln\left\{\frac{P_{\lambda }\left(X_{k}\right)}{P_{\lambda_{0}}\left(X_{k}\right)}\right\}=\left[X_{k}\ln\frac{\lambda }{\lambda_{0}}+\lambda_{0}-\lambda \right] \end{array}$$

So we define a single upward CUSUM chart as

(4) $$\begin{array}{c} T_{\mu_{i}}=\min\left\{n:\underset{1\leq k\leq n}{\max}\overset{n}{\underset{j=n-k+1}{\sum }}\;\left[X_{k}\ln\frac{\mu_{i}}{\lambda_{0}}+\lambda_{0}-\mu_{i}\right]>d_{i}\right\} \end{array}$$

where *d*_*i*_ is the width of the control limit and *μ*_*i*_ are some reference values satisfying *λ*_0_ = *a* < *μ*_1_ < *μ*_2_ < , ⋯, <*μ*_*m*_ ≤ *b*. We assumed that the possible range of the rate parameter shifts is (*a*, *b*).

Let Δ_*m*_ = {*μ*_*i*_ : 1 ≤ *i* ≤ *m*} and *W*_*m*_ = {*w*_*i*_ : 1 ≤ *i* ≤ *m*} be a set of numbers (known reference values) where *μ*_*i*_ > 1, *m* ≥ 2, and 0 < *w*_*i*_ ≤ 1. Also, let *D*_*m*_ = {*d*_*i*_ : 1 ≤ *i* ≤ *m*} and *H*_*m*_ = {*h*_*i*_ : 1 ≤ *i* ≤ *m*} be a set of numbers (width of control limit) where *d*_*i*_ > 0 which usually depends on *μ*_*i*_ and *h*_*i*_ > 0 also depends on *w*_*i*_.

We define a single upward exponential weighted moving average (EWMA) chart as

(5) $$\begin{array}{c} T_{E_{i}}\left(w_{i},h_{i}\right)=\min\left\{n:\overset{n-1}{\underset{k=0}{\sum }}\;w_{i}{\left(1-w_{i}\right)}^{k}X_{n-k}>h_{i}\right\} \end{array}$$

Let us define the one-sided CUSUM and EWMA multicharts as *T*_*CM*_(Δ_*m*_, *D*_*m*_) and *T*_*EM*_(*W*_*m*_, *H*_*m*_), respectively, where

(6) $$\begin{array}{c} \;T_{CM}\left(\Delta_{m},D_{m}\right)=\underset{\mu_{i}\in \Delta_{m}}{\min}\left\{T_{\mu_{i}}\left(\mu_{i},d_{i}\right)\right\} \end{array}$$

(7) $$\begin{array}{c} T_{EM}\left(W_{m},H_{m}\right)=\underset{1\leq i\leq m}{\min}\left\{T_{E_{i}}\left(w_{i},h_{i}\right)\right\} \end{array}$$

We also define the one-sided EWMA-CUSUM mixed charts as *T*_*EC*_ where

(8) $$\begin{array}{c} T_{EC}=\underset{1\leq i\leq m}{\min}\left\{T_{E_{i}}\left(w_{i},h_{i}\right)\;\text{or }T_{\mu_{i}}\left(\mu_{i},d_{i}\right)\right\} \end{array}$$

### 2.2. Charting Performance Index

The most widely used measure to determine which control chart performs better is the average run length (ARL). Ultimately, we force all the charts to have the same in-control average run length *ARL*_0_ then for a desired shift in the parameter of interest, the chart with the lowest out-of-control average run length (*ARL*_*λ*_) has the greatest ability to determine the prespecified shift. The ARL used in evaluating chart performance is weak due to the fact that its performance will deteriorate if the actual size of a mean shift is significantly different from the assumed size. To help address this problem, a number of novel charting performance indices have been proposed in the literature. For example, Han et al. [39] proposed the Overall Charting Performance Index (OCPI). Other charting performance measures include but not limited to Relative Mean Index (RMI) [42], Charting Performance Index (CPI) [43], etc. In the computation of these indices, we need the optimal *ARL* (*ARL*^∗^) which is found subject to normal distribution (continuous distribution). The CUSUM *ARLs* with reference values as charting statistic subject to Poisson distribution are not optimal as we are dealing with discrete distribution; hence, we also propose new measures (called Expectation of the Time for Detecting mean shifts (*ETD*) and Expectation of the Time for Detecting mean shifts with Equal weights of shifts (*ETDE*)) as performance index measures to evaluate the efficiency of the schemes.

We define the *ETD* of a chart (*T*) for a range of shifts in the rate parameter (*a*, *b*) by

(9) $$\begin{array}{c} ETD\left(T\right)=\overset{l}{\underset{i=1}{\sum }}\;w_{i}ARL_{\lambda_{i}}\left(T\right) \end{array}$$

where *w*_*i*_ = *λ*_*i*_/∑_*j*=1_^*l*^ *λ*_*j*_, *λ*_1_ < *λ*_2_ < ⋯<*λ*_*l*_ are real rate parameters and *l* is the number of shifts considered in the study.

When *w*_*i*_ = 1/*l*, we consider

(10) $$\begin{array}{c} ETDE\left(T\right)=\frac{1}{l}\overset{l}{\underset{i=1}{\sum }}\;ARL_{\lambda_{i}}\left(T\right) \end{array}$$

where *ETDE*(*T*) is the expectation of the time for detecting mean shifts when the *ARL*_*λ*_*i*__ are assigned equal weights of the inverse of the number of shifts considered in the study. Different forms of the weights can be studied, but here, we restrict it to these two scenarios. The chart with the smallest *ETD* and *ETDE* performs better.

### 2.3. Comparison of the Multi-chart Schemes with Its Constituent Charts

Without loss of generality, let *P*_0_(·) and *E*_0_(·) represent the probability and expectation that there is no change in the rate parameter, respectively. Let *P*_*λ*_(·) and *E*_*λ*_(·) represent the probability and expectation when there is a change in the true rate parameter (*λ*) at change point *ν* = 1, respectively. Normally for a stopping time *T*, we use out-control average run length (*ARL*_*λ*_) to judge which chart is performing better. All the charts were designed with a common *ARL*_0_ and for a shift in the rate parameter; we adjudge a chart with smaller *ARL*_*λ*_ to be the best performing. Intuitively, we define *ARL*_0_(*T*) = *E*_0_(*T*) and *ARL*_*λ*_(*T*) = *E*_*λ*_(*T*). Let's also assume that the rate parameter *λ* ≥ 1 and we choose some reference values satisfying *λ*_0_ = *a* < *μ*_1_ < *μ*_2_ < ⋯<*μ*_*m*_ ≤ *b*, where *m* is the number of charts. Let *d*_1_, *d*_2_, ⋯, *d*_*m*_ be the width of the individual control limits. We take the multi-chart control limits; *d*_1_′, *d*_2_′, ⋯, *d*_*m*_′ such that *d*_*i*_′ > *d*_*i*_, for 1 ≤ *i* ≤ *m*.

We can compare the multi-chart (*T*_*CM*_) = *T*_*CM*_(*d*_1_′, *d*_2_′, ⋯, *d*_*m*_′) with its constituent charts *T*_*μ*_*i*__(*d*_*i*_). If we choose *d*_1_′, *d*_2_′, ⋯, *d*_*m*_′ according to the restrictions

(11) $$\begin{array}{c} L_{0}=ARL_{0}\left(T\left(\mu_{1},d_{1}\right)\right)\approx ARL_{0}\left(T\left(\mu_{2},d_{2}\right)\right)\approx \cdots \approx ARL_{0}\left(T\left(\mu_{m},d_{m}\right)\right). \end{array}$$

That is if we force the in-control average run lengths of all the single CUSUM charts to be approximately equal. Similarly, to construct EWMA multi-chart, we force the in-control average run lengths of all the single EWMA charts to be approximately equal.

**Preposition 1 .**

Under the condition (11) and for large *L*_0_, we have

(12) $$\begin{array}{c} ETD\left(T_{CM}\right)\leq ETD\left(T_{\mu_{i}}\right)\;\text{for }1\leq i\leq m \end{array}$$

(13) $$\begin{array}{c} ETDE\left(T_{CM}\right)\leq ETDE\left(T_{\mu_{i}}\right)\;\text{for }1\leq i\leq m \end{array}$$

By inequalities (12) and (13), CUSUM multi-chart has better detection performance than single CUSUM charts. The proofs of these prepositions are in the Appendix.

Usually, it is difficult to predetermine the exact size of the mean shift before it is detected. Instead, a range of shift sizes of interest could be considered. We can compare the performances of these single charts with the average of these charts. We define the average CUSUM chart, average EWMA chart and average EWMA-CUSUM chart respectively as

(14) $$\begin{array}{c} Average\;CUSUM\;Chart=\frac{1}{m}\overset{m}{\underset{i=1}{\sum }}\;ARL_{\lambda_{i}}\left(T_{\mu_{i}}\left(\mu_{i},d_{i}\right)\right) \end{array}$$

(15) $$\begin{array}{c} Average\;EWMA\;Chart=\frac{1}{m}\overset{m}{\underset{i=1}{\sum }}\;ARL_{\lambda_{i}}\left(T_{E_{i}}\left(w_{i},h_{i}\right)\right) \end{array}$$

(16) $$\begin{array}{c} Average\;EWMA-CUSUM\;Chart=\frac{1}{m}\overset{m}{\underset{i=1}{\sum }}\;ARL_{\lambda_{i}}\left(T_{EC}\right) \end{array}$$

### 2.4. Procedural Description of Multi-chart Schemes

This section provides a detailed description of the simulation procedure used for the computation of ARL at each shift (*λ*), computation of ETD and ETDE for the comparison of the charts. We used Monte Carlo simulations for the computation of the ARLs. Simulation analyses were carried for a 10,000-repetition experiment. We generally set the in-control rate parameter (*λ*) = 1.

#### 2.4.1. Computation of the CUSUM Multi-chart Statistic

1. Determine the number of charts to be used for the CUSUM multichart. Sparks [44] suggested that three or more single charts are needed to achieve an efficient multi-chart scheme
2. Determine the reference parameters *μ*_1_, *μ*_2_, ⋯, *μ*_*k*_
3. Generate a random sample of size 1 at each step (denoted by *X*_*k*_) from the Poisson distribution with the specified reference value
4. Determine the in-control (*ARL*_0_) of the single charts say *ARL*_0_ ≈ 200 or 500 and use Monte Carlo simulations to find the control limits (*d*_1_, *d*_2_, ⋯, *d*_*k*_) of the single charts using equation (4)
5. Normally to arrive at an in-control ARL of CUSUM multi-chart (*T*_*CM*_) of approximately 200 or 500, we had to choose the single charts to have approximately equal in-control *ARL*_0_ = *L*_0_. Set *L*_0_ and use step (4) to determine the control limits; (*d*_1_′, *d*_2_′, ⋯, *d*_*k*_′). Adjust *d*_*i*_′ until the in-control *ARL*_0_ of CUSUM multi-chart is arrived at
6. Compute the *ARLs* of the single charts and CUSUM multi-chart using charting statistic (4) and (6), respectively. Compute the *ARLs* of the average CUSUM chart by equation (14)
7. Compute the *ETD* and *ETDE* of the CUSUM charts, average CUSUM, and CUSUM multi-chart using equations (9) and (10)

#### 2.4.2. Computation of the EWMA Multi-chart Statistic

1. Determine the number of charts to be used for the EWMA multi-chart. Generally, for the sake of comparison, we use the same number of charts as in the CUSUM setting
2. Determine the smoothing parameters *w*_1_, *w*_2_, ⋯, *w*_*k*_
3. Generate a random sample of size 1 at each step from the Poisson distribution
4. Determine the in-control (*ARL*_0_) of the single charts say *ARL*_0_ ≈ 200 or 500 and use Monte Carlo simulations to find the control limits (*h*_1_, *h*_2_, ⋯, *h*_*k*_) of the single charts using equation (5)
5. Normally to arrive at an in-control ARL of EWMA multi-chart (*T*_*EM*_) of approximately 200 or 500, we had to choose the single charts to have approximately equal in-control (*ARL*_0_) = *L*_0_. Set *L*_0_ and use step (4) to determine the control limits; (*h*_1_′, *h*_2_′, ⋯, *h*_*k*_′). Adjust the *h*_*i*_′ until the in-control *AR*L_0_ of EWMA multi-chart is arrived at
6. Compute the *ARLs* of the single charts and EWMA multi-chart using charting statistics (5) and (7), respectively. Compute the *ARLs* of the average EWMA chart by equation (15)
7. Compute the *ETD* and *ETDE* of the single EWMA charts, average EWMA chart and EWMA multi-chart using equations (9) and (10)

#### 2.4.3. Computation of the EWMA-CUSUM Multi-chart Statistic

1. Determine the number of charts to be used for the EWMA-CUSUM multi-chart. Generally, for the sake of comparison, we use the same number of charts as in the EWMA and CUSUM setting
2. Determine the smoothing parameters *w*_1_, *w*_2_, ⋯, *w*_*k*_ and reference values *μ*_1_, *μ*_2_, ⋯, *μ*_*k*_
3. Generate a random sample of size 1 at each step from the Poisson distribution
4. Determine the in-control (*ARL*_0_) of the single charts say *ARL*_0_ ≈ 200 or 500 and use Monte Carlo simulations to find the control limits (*h*_1_, *h*_2_, ⋯, *h*_*k*_; *d*_1_, *d*_2_, ⋯, *d*_*k*_) of the single charts using charting statistics (5) and (4).
5. Normally to arrive at an in-control ARL of EWMA-CUSUM multi-chart (*T*_*EC*_) of approximately 200 or 500, we had to choose the single charts to have approximately equal in-control (*ARL*_0_) = *L*_0_. Set *L*_0_ and use step (4) to determine the control limits; (*h*_1_′, *h*_2_′, ⋯, *h*_*k*_′; *d*_1_′, *d*_2_′, ⋯, *d*_*k*_′). Adjust the *h*_*i*_′ and *d*_*i*_′ until the in-control *ARL*_0_ of EWMA-CUSUM multi-chart is arrived at
6. Compute the *ARLs* of the single charts and EWMA-CUSUM multi-chart using charting statistic (5) and (8), respectively. Compute the *ARLs* of the average EWMA-CUSUM chart by equation (16).
7. Compute the *ETD* and *ETDE* of the single EWMA-CUSUM charts and EWMA-CUSUM multi-chart using equations (9) and (10).

## 3. Simulation Results and Discussion

In this section, we shall present and discuss the numerical results of the CUSUM and multi-CUSUM chart in subsection 1, present and discuss results for EWMA and EWMA multi-chart in subsection 2, discuss results for EWMA-CUSUM multi-chart in subsection 3, and compare results in subsection 4.

### 3.1. Simulation Results of CUSUM and Multi-CUSUM Chart

Simulation analyses were carried out for a 10,000-repetition experiment. We analyzed the simulation results for ten mean shifts in the rate parameter (*λ*_1_ = 1.25, *λ*_2_ = 1.50, *λ*_3_ = 1.75, *λ*_4_ = 2.00, *λ*_5_ = 2.25, *λ*_6_ = 2.50, *λ*_7_ = 2.75, *λ*_8_ = 3.00, *λ*_9_ = 3.25, and *λ*_10_ = 3.50) with change point *ν* = 1 that is the first time there is signal or change. For comparison sake, the in-control *ARL*(*ARL*_0_) of all the charts were assumed to be equal and was taken to be 200 and 500, respectively. The reference values *μ*_*i*_ were chosen to be *μ*_1_ = 1.5, *μ*_2_ = 2.0, and *μ*_3_ = 2.5, where *μ*_1_ is termed as a small mean shift in the rate parameter, *μ*_2_ is a medium mean shift, and *μ*_3_ is a large mean shift in the rate parameter, respectively. The simulation results for the out-control average run length (*ARL*_*λ*_) of the Poisson CUSUM charts with parameter *μ*_1_, *μ*_2_, *μ*_3_, average CUSUM chart and multi-chart (*T*_*CM*_) were listed in column two, column three, column four, column five, and column six, respectively. The parameter *d*_*i*_ and *d*_*i*_′ are the width of the control limits for the single CUSUM charts and CUSUM multi-chart, respectively. We chose three separate CUSUM charts because as suggested by Sparks [40], three or more single charts are needed to achieve an efficient multi-chart scheme. The control limits were obtained using Monte Carlo simulations. To arrive at an in-control ARL of CUSUM multi-chart (*T*_*CM*_) of approximately 200, we had to choose *ARL*_0_(*T*_1_(*μ*_1_)) = 279.86 with *d*_1_′ = 2.914062, *ARL*_0_(*T*_2_(*μ*_2_)) = 280.86 with *d*_2_′ = 3.59375, and *ARL*_0_(*T*_3_(*μ*_3_)) = 280.62 with *d*_3_′ = 3.749023. Similarly, to arrive at an in-control ARL of CUSUM multi-chart (*T*_*CM*_) of approximately 500, we had to choose *ARL*_0_(*T*_1_(*μ*_1_)) = 740.57 with *d*_1_′ = 3.794189, *ARL*_0_(*T*_2_(*μ*_2_)) = 740.86 with *d*_2_′ = 4.47998, and *ARL*_0_(*T*_3_(*μ*_3_)) = 741.11 with *d*_3_′ = 4.744361. In other words, we force all the *ARL*_0_ of the single charts to be approximately equal to guarantee an *ARL*_0_ of multi-chart to be approximately 200 and 500, respectively.

Tables 1 and 2 show that each of the schemes has its merits and demerits over a range, and perhaps, it is conflicting to compare the charts in relation to the average run length (*ARL*). Ultimately, the *ETD* and *ETDE* enable us to compare the charts over the whole range of shifts. CUSUM multi-chart (*T*_*CM*_) has the smallest *ETD* and *ETDE* followed by CUSUM chart *T*_*μ*_2__(*d*_2_), *T*_*μ*_1__(*d*_1_), and *T*_*μ*_3__(*d*_3_), respectively, for *ARL*_0_ = 200. Also, CUSUM multi-chart(*T*_*CM*_) has the smallest *ETD* and *ETDE* for the range of shifts followed by chart *T*_*μ*_1__(*d*_1_), *T*_*μ*_2__(*d*_2_), and *T*_*μ*_3__(*d*_3_), respectively, for *ARL*_0_ = 500.

Each of the single CUSUM charts has its main strength. For example, *T*_1_(*μ*_1_) is tuned to detect small shifts of the rate parameter, and it is the fastest for detecting (*λ* = 1.25 and 1.50). Chart *T*_2_(*μ*_2_) is the fastest for detecting medium shifts in the rate parameter (*λ* = 2.00 and 2.25) while chart *T*_3_(*μ*_3_) is the fastest for detecting large shifts in the rate parameter (*λ* = 2.50,2.75,3.00,3.25 and 3.50). The CUSUM multi-chart is also faster in detecting shifts in the mean than the average of the three single CUSUM charts.

### 3.2. Simulation Results of EWMA and EWMA Multi-chart

Tables 3 and 4 show the simulation analyses for EWMA single charts and EWMA multi-chart for an *ARL*_0_ of 200 and 500, respectively. The simulation analyses for EWMA and EWMA multi-charts were carried out for a 10,000-repetition experiment. We analyzed the simulation results for ten mean shifts in the rate parameter (*λ*_1_ = 1.25, *λ*_2_ = 1.50, *λ*_3_ = 1.75, *λ*_4_ = 2.00, *λ*_5_ = 2.25, *λ*_6_ = 2.50, *λ*_7_ = 2.75, *λ*_8_ = 3.00, *λ*_9_ = 3.25, and *λ*_10_ = 3.50) with change point *ν* = 1 that is the first time there is signal or change. We chose values of the smoothing parameter *w*_*i*_ to be *w*_1_ = 0.1, *w*_2_ = 0.5, and *w*_3_ = 0.9. The simulation results for the out-control average run length (*ARL*_*λ*_) of the EWMA and EWMA multi-charts were listed on Tables 3 and 4, respectively. The parameter *h*_*i*_ and *h*_*i*_′ are the width of the control limits for the single EWMA charts and EWMA multi-chart, respectively. We chose three separate EWMA charts similar to the CUSUM simulation setting.

The control limits were obtained using Monte Carlo simulations. To arrive at an in-control ARL of EWMA multi-chart (*T*_*EM*_) of approximately 200, we had to choose *ARL*_0_(*T*_*E*_1__(*w*_1_)) = 360.65 with *h*_1_′ = 1.59916, *ARL*_0_(*T*_*E*_2__(*w*_2_)) = 360.27 with *h*_2_′ = 3.00625, and *ARL*_0_(*T*_*E*_3__(*w*_3_)) = 359.26 with *h*_3_′ = 4.51543. Similarly, to arrive at an in-control ARL of EWMA multi-chart (*T*_*EM*_) of approximately 500, we had to choose *ARL*_0_(*T*_*E*_1__(*w*_1_)) = 899.40 with *h*_1_′ = 1.711293, *ARL*_0_(*T*_*E*_2__(*w*_2_)) = 900.53 with *h*_2_′ = 3.00625, and *ARL*_0_(*T*_*E*_3__(*w*_3_)) = 900.22 with *h*_3_′ = 4.689937. In other words, we force all the *ARL*_0_ of the single charts to be approximately equal to guarantee an *ARL*_0_ of multi-chart to be approximately 200 and 500, respectively.

Tables 3 and 4 show that each of the schemes has its merits and demerits over a range, and perhaps, it is conflicting to compare the charts in relation to the average run length (*ARL*). We use the *ETD* and *ETDE* to compare the charts over the whole range of shifts. EWMA multi-chart (*T*_*EM*_) has the smallest *ETD* and *ETDE* followed by EWMA chart *T*_*E*_2__(0.5), *T*_*E*_3__(0.9), and *T*_*E*_1__(0.1), respectively, for *ARL*_0_ = 200. Also, EWMA multi-chart (*T*_*EM*_) has the smallest *ETD* and *ETDE* followed by chart *T*_*E*_1__(0.1), *T*_*E*_2__(0.5) and *T*_*E*_3__(0.9), respectively, for *ARL*_0_ = 500. The EWMA multi-chart is also faster in detecting shifts in the mean than the average of the three single EWMA charts.

### 3.3. Simulation Results of CUSUM-EWMA-CUSUM Charts

Tables 5–8 show the simulation analyses for EWMA-CUSUM single charts and EWMA-CUSUM multi-chart for an *ARL*_0_ of 200 and 500, respectively. The simulation analyses were carried out for a 10,000-repetition experiment. We analyzed the simulation results for ten mean shifts in the rate parameter (*λ*_1_ = 1.25, *λ*_2_ = 1.50, *λ*_3_ = 1.75, *λ*_4_ = 2.00, *λ*_5_ = 2.25, *λ*_6_ = 2.50, *λ*_7_ = 2.75, *λ*_8_ = 3.00, *λ*_9_ = 3.25, and *λ*_10_ = 3.50) with change point *ν* = 1 that is the first time there is signal or change. For comparison sake, the in-control *ARL*(*ARL*_0_) of all the charts were assumed to be equal and was taken to be 200 and 500, respectively. We considered one EWMA chart and two CUSUM charts. We chose values of the smoothing parameter *w*_*i*_, to be *w*_1_ = 0.1 and reference parameters*μ*_1_ = 1.5 and *μ*_2_ = 2.5 for *T*_*EC*_1__ and *w*_1_ = 0.1, *μ*_1_ = 1.5 and *μ*_2_ = 2 for *T*_*EC*_2__. The simulation results for the out-control average run length (*ARL*_*λ*_) of the EWMA-CUSUM multi-charts were listed on Tables 5–8, respectively.

The control limits were obtained using Monte Carlo simulations. To arrive at an in-control ARL of EWMA-CUSUM multi-chart (*T*_*EC*_1__) of approximately 200, we had to choose *ARL*_0_(*T*_*E*_1__(*w*_1_)) = 304.92 with *h*_1_′ = 1.575862, *ARL*_0_(*T*_1_(*μ*_1_)) = 304.31 with *d*_1_′ = 3.828079, and *ARL*_0_(*T*_2_(*μ*_2_)) = 305.31 with *d*_2_′ = 4.825815. Similarly, to arrive at an in-control ARL of EWMA-CUSUM multi-chart (*T*_*EC*_1__) of approximately 500, we had to choose *ARL*_0_(*T*_*E*_1__(*w*_1_)) = 819.18 with *h*_1_′ = 1.699961, *ARL*_0_(*T*_1_(*μ*_1_)) = 819.22 with *d*_1_′ = 3.900391, and *ARL*_0_(*T*_2_(*μ*_2_)) = 820.04 with *d*_2_′ = 4.825815.

Also, to arrive at an in-control ARL of EWMA-CUSUM multi-chart (*T*_*EC*_2__) of approximately 200, we had to choose A*RL*_0_(*T*_*E*_1__(*w*_1_)) = 270.8328 with *h*_1_′ = 1.559829, *ARL*_0_(*T*_1_(*μ*_1_)) = 269.995 with *d*_1_′ = 2.87793, and *ARL*_0_(*T*_2_(*μ*_2_)) = 270.5916 with *d*_2_′ = 3.545212. Similarly, to arrive at an in-control ARL of EWMA-CUSUM multi-chart (*T*_*EC*_1__) of approximately 500, we had to choose *ARL*_0_(*T*_*E*_1__(*w*_1_)) = 740.0857 with *h*_1_′ = 1.687119, *ARL*_0_(*T*_1_(*μ*_1_)) = 739.1194 with *d*_1_′ = 3.803393, and *ARL*_0_(*T*_2_(*μ*_2_)) = 740.4778 with *d*_2_′ = 4.482666.

In other words, we force all the *ARL*_0_ of the single charts to be approximately equal to guarantee an *ARL*_0_ of multi-chart to be approximately 200 and 500, respectively.

Tables 5–8 show that each of the schemes has its merits and demerits over a range, and perhaps, it is conflicting to compare the charts in relation to the average run length (*ARL*). We use the *ETD* and *ETDE* to compare the charts over the whole range of shifts. EWMA-CUSUM multi-chart (*T*_*EC*_1__) has the smallest *ETD* and *ETDE* and hence better detection performance than (*T*_*EC*_2__).

### 3.4. Comparison of Results

The CUSUM multi-chart is better on the whole in detecting various mean shifts in the rate parameter than EWMA multi-chart and EWMA-CUSUM multi-chart. Furthermore, the EWMA multi-chart is better on the whole in detecting various mean shifts than EWMA-CUSUM multi-chart. We subsequently used CUSUM multi-chart to monitor the real data. Also, the simulation results support the theoretical analysis.

### 3.5. An Illustration with Health Surveillance Data

We use monthly tuberculosis (TB) data (see Supplementary Materials) from the northern regional health directorate of the Ghana Health Service, spanning the period of 2010 to 2017 to illustrate the implementation of a multi-CUSUM scheme for monitoring health data. The tuberculosis data consists of mainly monthly cases of three types of tuberculosis, namely tuberculosis arthritis (TB arthritis), tuberculosis meningitis (TB meningitis), and tuberculosis miliary (TB miliary). Tuberculosis is basically an infectious disease caused by a bacterial microorganism called mycobacterium tuberculosis. The disease mostly affects the lungs but can affect or spread to other parts of the body as well. TB is contagious and normally spreads into the air through sneezing, talking, and coughing of a person with TB of the lungs or throat. Symptoms of TB in the lungs may include bad cough that lasts three weeks or longer, weight loss, loss of appetite, coughing up blood or mucus, weakness or fatigue, fever, and night sweats. TB can be deadly if it is not treated well. Normally patients can take antibiotics like rifampicin through the supervision of a medical doctor [45].

Tuberculosis (TB) is one of the top ten causes of death worldwide [46]. In 2017, there were more than 10 million cases of active TB which resulted in 1.6 million deaths including 0.3 million among people with HIV. New infections occur in about 1% of the population each year and about 25% of the world's population is thought to be infected with TB [42]. More than 95% of deaths occurred in developing countries, and more than 50% in India, China, Indonesia, Pakistan, and the Philippines [46]. In Ghana, the total cases of notified tuberculosis in 2017 were about 14,550 [47].

Tuberculosis arthritis is a joint inflammation caused by the invasion of the joint by tuberculosis bacilli that have migrated from a primary infection, usually in the chest. The most common joints affected include the wrists, ankles, knees, hips, and spine [45].

Tuberculosis meningitis is a disease that affects the tissues covering the brain and spinal cord. Tuberculosis meningitis is caused by mycobacterium tuberculosis. The bacterial spreads to the brain and spine from other parts of the body usually the lungs [45].

Miliary tuberculosis is another form of tuberculosis where the disease or infection spreads through the entire body. This type of tuberculosis is normally associated with people whose immune system has already been compromised. This is also caused by mycobacterium tuberculosis [45].

**Figure 1:**
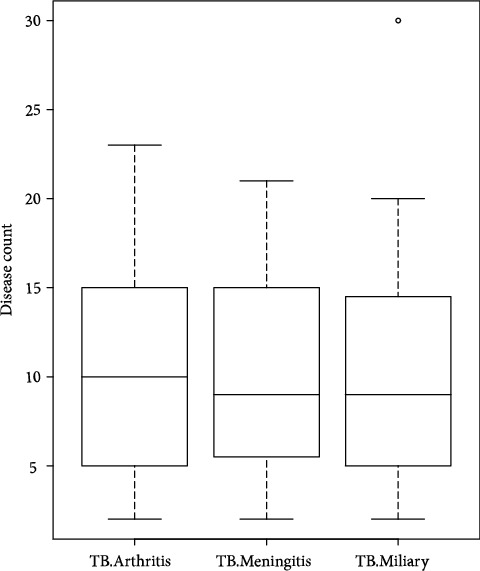
Box plot of count of TB arthritis, TB meningitis, and TB miliary between the years of 2010 to 2017.

**Figure 2:**
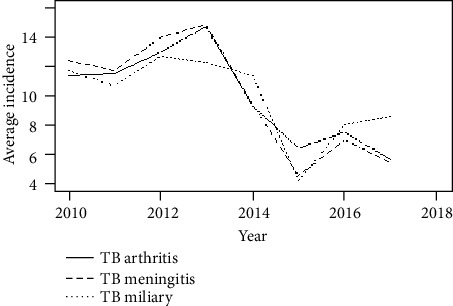
Annual average incidence of the diseases.

Figure 1 shows the box plot of the count of TB arthritis, TB meningitis, and TB miliary between the years of 2010 to 2017. The average count of TB Arthritis seems to be greater than the average of TB miliary and the average of TB meningitis. Also, the annual average incidence of the diseases varied from year to year as shown in (Figure 2). The means of the diseases seem to be dynamic, so we seek to detect changes in the average counts of the diseases. Many researchers have developed statistical methods for detecting changes in disease incidence or rates (see Mei et al. [28], Jiang et al. [29] and Richards et al. [30]). We proposed the multi-CUSUM chart for detecting changes in disease incidence. We consider the disease incidence as an *i*.*i*.*d*. random sequence, and we monitor the three tuberculosis diseases, namely tuberculosis arthritis, tuberculosis meningitis, and tuberculosis miliary. We applied the chi-square goodness-of-fit test to ascertain whether the data; {*Y*_*i*_ : *i* = 1, 2, ⋯, 96} is indeed coming from the Poisson distribution. The hypothesis of interest is *H*_0_: The form of the distribution for the data is Poisson; verses *H*_1_: The form of the distribution for the data is not Poisson. We control the test at a significance level of *α* = 0.05. If the *p* value of the goodness-of-fit test is greater than the specified significance level (*α*), we fail to reject *H*_0_ and conclude that the data is indeed Poisson *i*.*i*.*d*. We performed the chi-square goodness-of-fit test for the three diseases counts.

The p-values for the chi-square goodness-of-fit test are *p* value (TB arthritis) = 2.977376 × 10^−22^, *p* value (TB miliary) = 1.393786 × 10^−13^, and *p* value(TB meningitis) = 2.534261 × 10^09^. We therefore reject the assertion that the diseases are Poisson *i*.*i*.*d*.′*s* since the *p* values are less than the specified significance level. We consequently transformed the data by {$Y_{ij}/\sqrt{\lambda_{i}}:i=1,\cdots ,m$ and *j* = 1, 2, ⋯, 96}.

To detect changes in the tuberculosis diseases using the multi-CUSUM chart, we obtain estimates of the reference values of the diseases using data before 2013 as phase I data to estimate the rate parameters. The in-control reference values are *μ*_0*i*_ = (*μ*_01_, *μ*_02_, *μ*_03_), where say *μ*_01_ = median or mean of the in-control data, *μ*_02_ = third quarter of the in-control data, and *μ*_03_ = max of the in-control data; thus, we choose the reference values such that 1 < *μ*_01_ < *μ*_02_ < *μ*_03_. We then purpose to determine the detection capability of the multi-CUSUM chart to detect changes in the diseases starting from 2013.

We briefly expound the procedural steps for implementing the multi-CUSUM scheme for detecting changes in the tuberculosis disease incidence:

1. Standardized your observations {$Y_{ij}/\sqrt{\lambda_{i}}:i=1,\cdots ,m$ and *j* = 1, 2, ⋯, *n*}
2. Determine the in-control values of the reference parameters (*μ*_01_, *μ*_02_, *μ*_03_), Normally the in-control values are unknown; hence, we estimate them from the phase I historical data (e.g., estimate from data before 2013, say *μ*_01_ = median or mean of in-control data, *μ*_02_ = third quarter of in-control data, and *μ*_03_ = max of in-control data)
3. Determine the in-control (*ARL*_0_) of the individual charts say (*ARL*_0_) ≈ 50 and use Monte Carlo simulations to find the control limits of single charts
4. Assume some in-control (*ARL*_0_) of the multi-CUSUM charts say (*ARL*_0_) ≈ 50, then use Monte Carlo simulations to arrive at the control limits of the multi-CUSUM charts
5. Establish the multi-CUSUM chart then monitor new observations over time

Table 9 presents numerical results for monitoring TB arthritis, TB meningitis, and TB miliary starting from the 37th month (January 2013). According to Table 9, the in-control rate parameter (*λ*_0_) = 1. We use the same range of shifts in the rate parameter (*a*, *b*) and number of shifts (*l*) as in the simulation setting. The in-control *ARL*_0_ ≈ 50 means the expected number of false alarms for monitoring in phase II.

CUSUM multi-chart (*T*_*CM*_) was the quickest to detect changes in TB arthritis, since it has the smallest ETD and ETDE for detecting shifts in a range. Chart *T*_*μ*_2__(*d*_2_) also outperformed chart *T*_*μ*_3__(*d*_3_) and chart *T*_*μ*_1__(*d*_1_) in that order, respectively.

For TB meningitis and TB miliary disease, CUSUM multi-chart (*T*_*CM*_) was the quickest to detect changes, since it has the smallest ETD and ETDE for detecting shifts in a range followed by chart *T*_*μ*_3__(*d*_3_), *T*_*μ*_2__(*d*_2_), and *T*_*μ*_1__(*d*_1_) in that respective order. In general, CUSUM multi-chart is faster in detecting changes in the diseases (TB arthritis, TB meningitis, and TB miliary) than the single charts.

## 4. Conclusion

Basically, this study seeks to monitor tuberculosis diseases based on multi-chart schemes and also evaluate the efficiency of the proposed scheme. To achieve this purpose we carried out a simulation study for CUSUM multi-chart, EWMA multi-chart and EWMA-CUSUM multi-chart subject to the Poisson distribution. The chart with the smallest expectation of the time for detecting mean shifts (ETD) and the smallest expectation of the time for detecting mean shifts when the *ARL*_*λ*_*i*__ are assigned equal weights (ETDE) is better. The simulation results show that CUSUM multi-chart had the smallest ETD and ETDE; hence, CUSUM multi-chart has better detection performance than EWMA multi-chart and EWMA-CUSUM multi-chart. Also, the average of the CUSUM charts performed less better than CUSUM multi-chart likewise the average of the EWMA charts performed less better than EWMA multi-chart.

We subsequently used CUSUM multi-chart to monitor tuberculosis (TB) disease from the northern region of Ghana spanning the period of 2010 to 2017. The tuberculosis diseases were TB arthritis, TB meningitis, and TB miliary. We used the data before 2013 as phase 1 historical data for estimating reference values then starting from the 37th month (January 2013) we seek to monitor changes in disease incidence. CUSUM multi-chart (*T*_*CM*_) was the quickest to detect changes followed by chart T_*μ*_2__(*d*_2_), *T*_*μ*_3__(*d*_3_), and *T*_*μ*_1__(*d*_1_) in that order, respectively, for TB arthritis disease. For TB meningitis and TB miliary disease, CUSUM multi-chart (*T*_*CM*_) was the quickest to detect changes followed by chart *T*_*μ*_3__(*d*_3_), *T*_*μ*_2__(*d*_2_), and *T*_*μ*_1__(*d*_1_) in that respective order. Apparently, the size of shift in TB arthritis disease was medium, while the size of the shift was pretty large for TB meningitis and TB miliary.

Early detection of upward abrupt changes in the diseases could send warning signals to public health workers to trigger public awareness, education, and general control of tuberculosis in the northern region as well as other regions of Ghana. Further research can consider how the procedures considered in the article may be modified or adapted using nonparametric monitoring methods and also methods that will account for dependence among the diseases in case there are strong correlations among the diseases. Also, other distributions like the negative binomial can be considered to account for the possible effect of overdispersion.

## Figures and Tables

**Table 1:** ARLs with their SDRL (in bracket) for the CUSUM and multi-CUSUM chart with *ARL*_0_ ≈ 200.

Shifts in (*λ*)	*T*_1_(*μ*_1_)	*T*_2_(*μ*_2_)	*T*_3_(*μ*_3_)	Average CUSUM	*T*_*CM*_ (*μ*_1_, *μ*_2_, *μ*_3_)
	*d*_1_ = 2.609375	*d*_2_ = 3.238342	*d*_3_ = 3.453125		
1.00	202.53 (192.35)	204.21 (203.3)	203.86 (202.29)	203.53 (199.31)	200.91 (194.15)
1.25	45.44 (37.36)	51.29 (46.61)	58.45 (56.17)	51.73 (46.71)	48.11 (41.63)
1.50	20.64 (14.18)	21.77 (18.01)	24.25 (21.58)	22.22 (17.92)	20.90 (15.07)
1.75	12.79 (8.96)	12.67 (8.70)	13.50 (10.94)	12.99 (9.53)	12.61 (7.75)
2.00	9.17 (6.71)	8.71 (4.92)	8.95 (5.62)	8.94 (5.75)	8.85 (5.44)
2.25	7.26 (4.05)	6.59 (3.89)	6.71 (3.65)	6.85 (3.86)	6.74 (3.98)
2.50	6.03 (3.08)	5.41 (2.98)	5.25 (2.91)	5.56 (2.99)	5.47 (3.01)
2.75	5.15 (2.55)	4.51 (2.37)	4.29 (2.31)	4.65 (2.41)	4.60 (2.50)
3.00	4.47 (2.12)	3.93 (2.06)	3.72 (1.94)	4.04 (2.04)	3.91 (1.98)
3.25	4.01 (1.77)	3.51 (1.73)	3.27 (1.64)	3.60 (1.71)	3.45 (1.69)
3.50	3.59 (1.49)	3.15 (1.44)	2.92 (1.41)	3.22 (1.45)	3.07 (1.42)
ETD	8.971	8.898	9.389	9.086	8.692
ETDE	11.855	12.154	13.131	12.380	11.771

**Table 2:** ARLs with their SDRL (in bracket) for the CUSUM and multi-CUSUM chart with *ARL*_0_ ≈ 500.

Shifts in (*λ*)	*T*_1_(*μ*_1_)	*T*_2_(*μ*_2_)	*T*_3_(*μ*_3_)	Average CUSUM	*T*_*CM*_ (*μ*_1_, *μ*_2_, *μ*_3_)
	*d*_1_ = 3.430471	*d*_2_ = 4.090408	*d*_3_ = 4.331055		
1.00	500.54 (490.24)	501.72 (492.04)	501.17 (495.91)	501.14 (492.73)	500.17 (485.63)
1.25	70.77 (58.68)	88.97 (83.20)	102.39 (99.86)	87.38 (80.58)	75.69 (63.93)
1.50	27.66 (18.25)	30.87 (24.72)	36.28 (32.23)	31.60 (25.07)	28.81 (19.96)
1.75	16.54 (9.14)	16.75 (11.80)	18.06 (14.48)	17.12 (11.81)	16.41 (10.24)
2.00	11.79 (5.67)	10.99 (6.84)	11.45 (8.25)	11.41 (6.92)	11.23 (6.53)
2.25	9.28 (4.13)	8.13 (4.45)	8.02 (5.12)	8.48 (4.57)	8.51 (4.80)
2.50	7.55 (3.25)	6.48 (3.38)	6.30 (3.79)	6.78 (3.47)	6.68 (3.65)
2.75	6.48 (2.65)	5.42 (2.70)	5.14 (2.79)	5.68 (2.71)	5.51 (2.86)
3.00	5.65 (2.23)	4.71 (2.23)	4.39 (2.24)	4.92 (2.23)	4.69 (2.40)
3.25	5.03 (1.90)	4.10 (1.89)	3.89 (1.90)	4.34 (1.90)	4.05 (2.02)
3.50	4.52 (1.65)	3.65 (1.61)	3.46 (1.61)	3.88 (1.62)	3.60 (1.75)
ETD	12.176	12.566	13.590	12.779	11.783
ETDE	16.527	18.007	19.938	18.159	16.518

**Table 3:** ARLs with their SDRL (in bracket) for the EWMA chart and EWMA multichart with *ARL*_0_ ≈ 200.

Shifts in (*λ*)	*T*_*E*_1__(0.1)	*T*_*E*_2__(0.5)	*T*_*E*_3__(0.9)	Average EWMA	*T*_*EM*_
	*h*_1_ = 1.517578	*h*_2_ = 2.815918	*h*_3_ = 3.806445		
1.00	201.51 (180.30)	200.89 (198.31)	199.37 (194.44)	200.59 (191.02)	201.30 (191.34)
1.25	51.92 (35.82)	66.89 (63.94)	76.04 (76.97)	64.95 (58.91)	54.83 (44.98)
1.50	27.19 (14.17)	30.43 (27.86)	35.04 (34.94)	30.89 (25.66)	25.93 (18.08)
1.75	18.47 (8.01)	17.20 (14.83)	19.79 (18.99)	18.49 (13.94)	16.22 (10.57)
2.00	14.05 (5.34)	11.10 (8.85)	12.25 (11.33)	12.47 (8.51)	11.30 (7.25)
2.25	11.39 (3.98)	7.94 (5.98)	8.47 (7.62)	9.27 (5.86)	8.57 (5.49)
2.50	9.69 (3.15)	6.14 (4.36)	6.34 (5.45)	7.39 (4.32)	6.74 (4.36)
2.75	8.41 (2.63)	4.94 (3.15)	4.90 (4.04)	6.08 (3.27)	5.49 (3.53)
3.00	7.48 (2.23)	4.13 (2.58)	3.97 (3.17)	5.19 (2.66)	4.59 (2.93)
3.25	6.74 (1.96)	3.58 (2.16)	3.31 (2.48)	4.54 (2.20)	3.97 (2.48)
3.50	6.13 (1.75)	3.15 (1.81)	2.88 (2.05)	4.05 (1.87)	3.42 (2.08)
ETD	12.837	11.091	12.121	12.016	10.454
ETDE	16.147	15.550	17.299	16.332	14.106

**Table 4:** ARLs with their SDRL (in bracket) for the EWMA charts and EWMA multichart with *ARL*_0_ ≈ 500.

Shifts in (*λ*)	*T*_*E*_1__(0.1)	*T*_*E*_2__(0.5)	*T*_*E*_3__(0.9)	Average EWMA	*T*_*EM*_
	*h*_1_ = 1.641053	*h*_2_ = 3.102122	*h*_3_ = 4.599133		
1.00	500.94 (484.69)	501.15 (500.08)	501.23 (496.67)	501.11 (493.81)	501.97 (492.12)
1.25	82.02 (61.84)	138.95 (136.88)	163.51 (162.92)	128.16 (120.55)	95.57 (82.52)
1.50	35.52 (20.29)	54.14 (53.46)	72.77 (71.59)	54.14 (48.45)	37.43 (25.96)
1.75	22.22 (10.15)	27.86 (25.25)	37.67 (36.63)	29.25 (24.01)	21.23 (13.16)
2.00	16.54 (6.44)	16.56 (14.17)	22.99 (21.50)	18.70 (14.04)	14.37 (8.78)
2.25	13.04 (4.67)	11.19 (8.98)	14.90 (13.57)	13.04 (9.07)	10.47 (6.36)
2.50	10.82 (3.54)	8.14 (6.07)	10.61 (9.44)	9.86 (6.35)	8.17 (5.14)
2.75	9.44 (2.96)	6.31 (4.46)	7.99 (6.95)	7.91 (4.79)	6.52 (4.19)
3.00	8.27 (2.48)	5.19 (3.40)	6.11 (5.02)	6.52 (3.63)	5.37 (3.44)
3.25	7.47 (2.19)	4.36 (2.75)	5.13 (4.02)	5.65 (2.99)	4.54 (2.90)
3.50	6.76 (1.91)	3.78 (2.23)	4.20 (3.15)	4.91 (2.43)	3.92 (2.48)
ETD	16.121	18.637	23.458	19.404	**14.653**
ETDE	21.210	27.648	34.588	27.814	**20.759**

**Table 5:** ARLs with their SDRL (in bracket) for the CUSUM-EWMA-CUSUM charts with *ARL*_0_ ≈ 200.

Shifts in (*λ*)	*T*_*E*_1__(0.1)	*T*_1_(*μ*_1_ = 1.5)	*T*_2_(*μ*_2_ = 2.5)	Average EWMA-CUSUM	*T*_*EC*_1__
	*h*_1_ = 1.517578	*d*_1_ = 2.609375	*d*_2_ = 3.453125		
1.00	201.51 (180.30)	202.53 (192.35)	203.86 (202.29)	202.63 (191.65)	200.20 (190.23)
1.25	51.92 (35.82)	45.44 (37.36)	58.45 (56.17)	51.94 (43.12)	47.14 (39.18)
1.50	27.19 (14.17)	20.64 (14.18)	24.25 (21.58)	24.03 (16.64)	21.66 (15.37)
1.75	18.47 (8.01)	12.79 (8.96)	13.50 (10.94)	14.92 (9.30)	13.15 (8.58)
2.00	14.05 (5.34)	9.17 (6.71)	8.95 (5.62)	10.72 (5.89)	9.19 (5.66)
2.25	11.39 (3.98)	7.26 (4.05)	6.71 (3.65)	8.45 (3.89)	7.08 (4.21)
2.50	9.69 (3.15)	6.03 (3.08)	5.25 (2.91)	6.99 (3.05)	5.57 (3.22)
2.75	8.41 (2.63)	5.15 (2.55)	4.29 (2.31)	5.95 (2.50)	4.66 (2.65)
3.00	7.48 (2.23)	4.47 (2.12)	3.72 (1.94)	5.22 (2.10)	3.96 (2.19)
3.25	6.74 (1.96)	4.01 (1.77)	3.27 (1.64)	4.67 (1.79)	3.46 (1.90)
3.50	6.13 (1.75)	3.59 (1.49)	2.92 (1.41)	4.21 (1.55)	3.11 (1.62)
ETD	12.837	8.971	9.389	10.398	8.821
ETDE	16.147	11.855	13.131	13.710	11.898

**Table 6:** ARLs with their SDRL (in bracket) for the CUSUM-EWMA-CUSUM charts with *ARL*_0_ ≈ 500.

Shifts in (*λ*)	*T*_*E*_1__(0.1)	*T*_1_(*μ*_1_ = 1.5)	*T*_2_(*μ*_2_ = 2.5)	Average EWMA-CUSUM	*T*_*EC*_1__
	*h*_1_ = 1.641053	*d*_1_ = 3.794189	*d*_2_ = 4.744361		
1.00	500.94 (484.69)	500.54 (490.24)	501.17 (495.91)	500.88 (490.28)	501.78 (486.69)
1.25	82.02 (61.84)	70.77 (58.68)	102.39 (99.86)	85.06 (73.46)	76.35 (62.88)
1.50	35.52 (20.29)	27.66 (18.25)	36.28 (32.23)	33.15 (23.59)	29.13 (19.82)
1.75	22.22 (10.15)	16.54 (9.14)	18.06 (14.48)	18.94 (11.26)	17.05 (10.40)
2.00	16.54 (6.44)	11.79 (5.67)	11.45 (8.25)	13.26 (6.79)	11.52 (6.74)
2.25	13.04 (4.67)	9.28 (4.13)	8.02 (5.12)	10.11 (4.64)	8.67 (5.04)
2.50	10.82 (3.54)	7.55 (3.25)	6.30 (3.79)	8.22 (3.53)	6.75 (3.78)
2.75	9.44 (2.96)	6.48 (2.65)	5.14 (2.79)	7.02 (2.80)	5.54 (2.99)
3.00	8.27 (2.48)	5.65 (2.23)	4.39 (2.24)	6.10 (2.32)	4.71 (2.44)
3.25	7.47 (2.19)	5.03 (1.90)	3.89 (1.90)	5.46 (2.00)	4.12 (2.10)
3.50	6.76 (1.91)	4.52 (1.65)	3.46 (1.61)	4.91 (1.72)	3.65 (1.77)
ETD	16.121	12.176	13.590	13.960	11.955
ETDE	21.21	16.527	19.938	19.223	16.749

**Table 7:** ARLs with their SDRL (in bracket) for the CUSUM-EWMA-CUSUM charts with *ARL*_0_ ≈ 200.

Shifts in (*λ*)	*T*_*E*_1__(0.1)	*T*_1_(*μ*_1_ = 1.5)	*T*_2_(*μ*_2_ = 2.0)	Average EWMA-CUSUM	*T*_*EC*_2__
	*h*_1_ = 1.517578	*d*_1_ = 2.609375	*d*_2_ = 3.453125		
1.00	201.51 (180.30)	202.53 (192.35)	204.21 (203.3)	202.75 (191.98)	199.58 (192.24)
1.25	51.92 (35.82)	45.44 (37.36)	51.29 (46.61)	49.55 (39.93)	46.76 (38.31)
1.50	27.19 (14.17)	20.64 (14.18)	21.77 (18.01)	23.20 (15.45)	21.45 (15.34)
1.75	18.47 (8.01)	12.79 (8.96)	12.67 (8.70)	14.64 (8.56)	12.80 (8.03)
2.00	14.05 (5.34)	9.17 (6.71)	8.71 (4.92)	10.64 (5.66)	9.18 (5.46)
2.25	11.39 (3.98)	7.26 (4.05)	6.59 (3.89)	8.41 (3.97)	7.00 (3.86)
2.50	9.69 (3.15)	6.03 (3.08)	5.41 (2.98)	7.04 (3.07)	5.76 (3.10)
2.75	8.41 (2.63)	5.15 (2.55)	4.51 (2.37)	6.02 (2.52)	4.84 (2.41)
3.00	7.48 (2.23)	4.47 (2.12)	3.93 (2.06)	5.29 (2.14)	4.20 (2.02)
3.25	6.74 (1.96)	4.01 (1.77)	3.51 (1.73)	4.75 (1.82)	3.70 (1.68)
3.50	6.13 (1.75)	3.59 (1.49)	3.15 (1.44)	4.29 (1.56)	3.35 (1.47)
ETD	12.837	8.971	8.898	10.233	8.892
ETDE	16.147	11.855	12.154	13.383	11.904

**Table 8:** ARLs with their SDRL (in bracket) for the CUSUM-EWMA-CUSUM charts with *ARL*_0_ ≈ 500.

Shifts in (*λ*)	*T*_*E*_1__(0.1)	*T*_1_(*μ*_1_ = 1.5)	*T*_2_(*μ*_2_ = 2.0)	Average EWMA-CUSUM	*T*_*EC*_2__
	*h*_1_ = 1.641053	*d*_1_ = 3.430471	*d*_2_ = 4.090408		
1.00	500.94 (484.69)	500.54 (490.24)	501.72 (492.04)	501.07 (488.99)	501.25 (492.97)
1.25	82.02 (61.84)	70.77 (58.68)	88.97 (83.2)	80.59 (67.91)	74.70 (62.12)
1.50	35.52 (20.29)	27.66 (18.25)	30.87 (24.72)	31.35 (21.09)	29.24 (19.67)
1.75	22.22 (10.15)	16.54 (9.14)	16.75 (11.80)	18.50 (10.36)	16.84 (10.09)
2.00	16.54 (6.44)	11.79 (5.67)	10.99 (6.84)	13.11 (6.32)	11.65 (6.54)
2.25	13.04 (4.67)	9.28 (4.13)	8.13 (4.45)	10.15 (4.42)	8.79 (4.68)
2.50	10.82 (3.54)	7.55 (3.25)	6.48 (3.38)	8.28 (3.39)	7.02 (3.50)
2.75	9.44 (2.96)	6.48 (2.65)	5.42 (2.70)	7.11 (2.77)	5.86 (2.83)
3.00	8.27 (2.48)	5.65 (2.23)	4.71 (2.23)	6.21 (2.31)	5.04 (2.35)
3.25	7.47 (2.19)	5.03 (1.90)	4.10 (1.89)	5.53 (1.99)	4.44 (2.00)
3.50	6.76 (1.91)	4.52 (1.65)	3.65 (1.61)	4.98 (1.72)	3.98 (1.72)
ETD	16.121	12.176	12.566	13.620	12.081
ETDE	21.210	16.527	18.007	18.581	16.756

**Table 9:** CUSUM and Multi-CUSUM chart results for monitoring TB arthritis, TB meningitis, and TB miliary with *ARL*_0_ ≈ 50.

Control chart	*T*_1_(*μ*_1_)	*T*_2_(*μ*_2_)	*T*_3_(*μ*_3_)	*T*_*CM*_(*μ*_1_, *μ*_2_, *μ*_3_)
TB arthritis				
Control limit	*d*_1_ = 0.72	*d*_2_ = 1.54	*d*_3_ = 1.1919	*d*′ = (0.8367187,1.75,2.28125)
Reference values	*μ*_01_ = 1.1609	*μ*_02_ = 1.5142	*μ*_03_ = 2.1199	
ETD	5.512	5.119	5.122	5.071
ETDE	6.832	6.443	6.556	6.405
TB meningitis				
Control limit	*d*_1_ = 1.046875	*d*_2_ = 1.657812	*d*_3_ = 1.964062	*d*′ = (1.1375,1.817188,2.179652)
Reference values	*μ*_01_ = 1.2632	*μ*_02_ = 1.6168	*μ*_03_ = 2.0211	
ETD	5.254	5.175	5.085	5.041
ETDE	6.542	6.537	6.478	6.372
TB miliary				
Control limit	*d*_1_ = 0.7148438	*d*_2_ = 1.504687	*d*_3_ = 2.354688	*d*′ = (0.9222656,1.889893,2.591463)
Reference values	*μ*_01_ = 1.1597	*μ*_02_ = 1.5126	*μ*_03_ = 3.0252	
ETD	5.505	5.105	4.954	4.878
ETDE	6.805	6.420	6.370	6.228

## Data Availability

The data used to support the findings of this study are available from the corresponding author upon request.

## Appendix

### Proofs of Theorems and Propositions

Proof of Proposition 1. For any real mean *λ*_*j*_ where 1 ≤ *j* ≤ *l*, we can take some reference value *μ*_*i*_0__ depending on *λ*_*j*_ such that

(A.1) $$\begin{array}{c} \left[\lambda_{j}\ln\mu_{i_{0}}+1-\mu_{i_{0}}\right]>\left[\lambda_{j}\ln\mu_{i}+1-\mu_{i}\right]\vee 0 \end{array}$$

for all *i* ≠ *i*_0_, where *x*∨0 denotes max(*x*, 0). It follows from Theorem 3.1 and Lemma 3.2 in Han and Tsung [43] that for large *L*_0_ we have

(A.2) $$\begin{array}{c} d_{j}^{\prime }/d_{j}\approx 1, \end{array}$$

(A.3) $$\begin{array}{c} E_{\lambda_{j}}\left(T_{\mu_{i}}\right)\approx \frac{d_{j}}{\left[\lambda_{j}\ln\mu_{i}+1-\mu_{i}\right]\vee 0} \end{array}$$

for 1 ≤ *j* ≤ *l* and 1 ≤ *i* ≤ *m*, where *λ*_0_ = 1. Hence, by (A.1), (A.2), and (A.3), we have

(A.4) $$\begin{array}{c} ETD\left(T_{CM}\right)=\overset{l}{\underset{j=1}{\sum }}\;{\bar{\lambda }}_{j}E_{\lambda_{j}}\left(T_{CM}\right)\leq \overset{l}{\underset{j=1}{\sum }}\;{\bar{\lambda }}_{j}E_{\lambda_{j}}\left(T_{\mu_{i}}\right)\approx ETD\left(T_{\mu_{i}}\right) \end{array}$$

for 1 ≤ *i* ≤ *m*, where ${\bar{\lambda }}_{j}=\lambda_{j}/\sum_{k=1}^{l}\;\lambda_{k}$ and $\sum_{j=1}^{l}\;{\bar{\lambda }}_{j}=1$ for 1 ≤ *j* ≤ *l*. This proves (12). Similarly, we can prove (13).

## Supplementary Materials

Supplementary Materials Real data to support simulation studies for subsection 3.2.
